# Landscape-Scale Controls on Aboveground Forest Carbon Stocks on the Osa Peninsula, Costa Rica

**DOI:** 10.1371/journal.pone.0126748

**Published:** 2015-06-10

**Authors:** Philip Taylor, Gregory Asner, Kyla Dahlin, Christopher Anderson, David Knapp, Roberta Martin, Joseph Mascaro, Robin Chazdon, Rebecca Cole, Wolfgang Wanek, Florian Hofhansl, Edgar Malavassi, Braulio Vilchez-Alvarado, Alan Townsend

**Affiliations:** 1 The Institute of Artic and Alpine Research, University of Colorado, Boulder, CO, 80303, United States of America; 2 Department of Global Ecology, Carnegie Institution for Science, Stanford, CA, 94305, United States of America; 3 National Center for Atmospheric Research, Advanced Study Program, Boulder, Colorado, 80307, United States of America; 4 Department of Ecology and Evolutionary Biology, University of Connecticut, Storrs, Connecticut, 06269, United States of America; 5 Department of Microbiology and Ecosystem Science, University of Vienna, Vienna, 1010, Austria; 6 Instituto Tecnológico de Costa Rica, Escuela de Ingenieria Forestal, Cartago, Costa Rica; Chinese Academy of Sciences, CHINA

## Abstract

Tropical forests store large amounts of carbon in tree biomass, although the environmental controls on forest carbon stocks remain poorly resolved. Emerging airborne remote sensing techniques offer a powerful approach to understand how aboveground carbon density (ACD) varies across tropical landscapes. In this study, we evaluate the accuracy of the Carnegie Airborne Observatory (CAO) Light Detection and Ranging (LiDAR) system to detect top-of-canopy tree height (TCH) and ACD across the Osa Peninsula, Costa Rica. LiDAR and field-estimated TCH and ACD were highly correlated across a wide range of forest ages and types. Top-of-canopy height (TCH) reached 67 m, and ACD surpassed 225 Mg C ha^-1^, indicating both that airborne CAO LiDAR-based estimates of ACD are accurate in tall, high-biomass forests and that the Osa Peninsula harbors some of the most carbon-rich forests in the Neotropics. We also examined the relative influence of lithologic, topoedaphic and climatic factors on regional patterns in ACD, which are known to influence ACD by regulating forest productivity and turnover. Analyses revealed a spatially nested set of factors controlling ACD patterns, with geologic variation explaining up to 16% of the mapped ACD variation at the regional scale, while local variation in topographic slope explained an additional 18%. Lithologic and topoedaphic factors also explained more ACD variation at 30-m than at 100-m spatial resolution, suggesting that environmental filtering depends on the spatial scale of terrain variation. Our result indicate that patterns in ACD are partially controlled by spatial variation in geologic history and geomorphic processes underpinning topographic diversity across landscapes. ACD also exhibited spatial autocorrelation, which may reflect biological processes that influence ACD, such as the assembly of species or phenotypes across the landscape, but additional research is needed to resolve how abiotic and biotic factors contribute to ACD variation across high biomass, high diversity tropical landscapes.

## Introduction

Tropical forests play an important role in the global carbon cycle by absorbing and storing atmospheric CO_2_ in tree biomass[1, 2]. Altogether they contain between 158 and 354 Pg C in forest biomass [3], which equates to 17 to 40 times the current annual rate of global fossil fuel emissions. However, tropical carbon stocks may be diminishing through deforestation and forest degradation [4,5], and potentially from increases in atmospheric temperature and drought severity [6]. With rising pressures on forests globally, it is important to understand patterns of and controls over forest biomass and dynamics, particularly in regions where terrestrial CO_2_ uptake and storage may be very high, such in the humid tropics.

Fertile lowland tropical forests remain understudied compared to other regions where older soils impoverished of bedrock nutrients are thought to limit tropical forest productivity, like the Eastern and Central Amazon [7]. Moreover, vast areas throughout the Western Amazon [2], Mesoamerica [8] and Southeast Asia [9] often display comparatively higher levels of forest productivity and carbon storage than do their temperate counterparts. While these more fertile regions may play a disproportionate role in terrestrial CO_2_ uptake and storage, the spatial variation in, and controls over, aboveground carbon density (ACD) remain poorly understood.

Much of the uncertainty reflects inadequate measurement of forest biomass across large unexplored areas. Traditionally, field inventory plots have been used to estimate tropical forest carbon storage (e.g. [10]), and such plot networks have informed our understanding of processes affecting tropical forest structure and function (e.g. [6]). Yet field approaches are typically conducted at or less than one hectare in size, and even with the advent of large-scale, long-term monitoring networks, field-based approaches cover a minuscule proportion of tropical land area and/or they involve plots that are not randomly or systematically arrayed across the land surface. Such spatial limitations constrain the ability of field efforts to capture the diversity of rainforests, and associated carbon storage, across the tremendous phylogenetic and biogeochemical heterogeneity of the tropics [11]. This heterogeneity emerges at scales ranging from sub-hectare to thousands of square kilometers, requiring data that has both high local fidelity and large regional coverage.

Emerging techniques in remote sensing can provide such multi-scale resolution [12, 13], and offer a complimentary approach to field inventory data. Airborne Light Detection and Ranging (LiDAR), which can be used to derive metrics of forest structure by emitting laser pulses (e.g. [14, 15, 16]), can be flown over thousands of hectares of tropical forest per day to produce spatially continuous estimates of ACD at levels of uncertainty approaching that which is achieved in field inventory plots [17, 18]. Importantly, airborne approaches provide information at higher spatial resolution than do current or forthcoming satellite systems. Moreover, the limited sensitivity of current satellite sensors to variation in forest structure in mature tropical forests greatly reduces the ability of current algorithms to accurately estimate ACD ([19]. Fortunately, high-resolution maps of airborne LiDAR-estimated ACD can be paired with environmental data to understand the fundamental abiotic and biotic controls over ACD [20, 21], which is important for predicting how tropical carbon stocks may change in the future.

**Fig 1:**
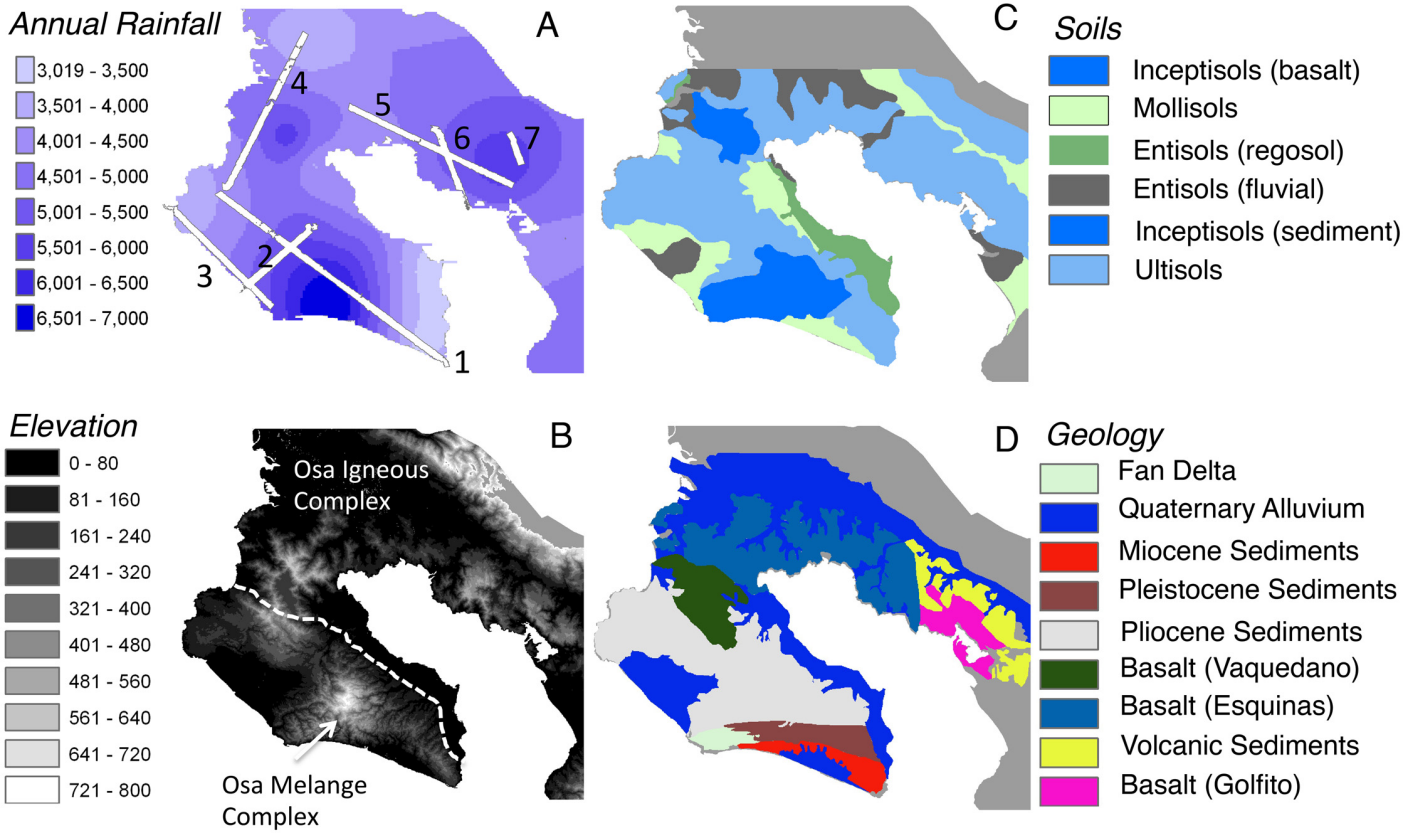
Environmental gradients across the Osa Peninsula, Costa Rica. (A) Distribution of precipitation ranging from 3000–7000 mm annually. White lines are LiDAR flight lines of the Carnegie Airborne Observatory. (B) Elevation based on SRTM data at 90 m resolution. (C) Soil order. (D) Geologic substrate.

Here we examine patterns in and environmental controls on ACD across the Osa Peninsula in southwest Costa Rica. The peninsula is comprised of basaltic and sedimentary substrates, and it is undergoing rapid geologic uplift (2.5–6 m k yr^-1^), combined with strong erosional weathering due to high levels of rainfall (3.5–7 m yr^-1^). These geomorphic forces have given rise to a generally fertile region that harbors wide gradients and diverse combinations of lithological, topoedaphic and climatic factors (Fig 1). Using the Carnegie Airborne Observatory (CAO][16], we evaluate the ability of airborne LiDAR to estimate tropical forest ACD and then examine landscape controls on ACD patterns in areas not directly and recently affected by human land use. Our study includes very high biomass forests with trees that extend over 60 m in height—two conditions that have not been tested well with airborne LiDAR or with methods for converting LiDAR-based measurements of forest structure to field-based estimates of aboveground biomass.

## Materials and Methods

### Study region

Much of the Osa Peninsula is protected, including Corcovado and Piedras Blancas National Parks. This region constitutes the largest tract of lowland forest on the American Pacific coastline. The region harbors high biogeochemical and biological diversity, largely due to a varied geologic history. The Osa Peninsula is at the convergent margin between Central America and subducting Cocos Plate, which originated at the Galapagos paleo-hotspot, and is currently moving northwest at a rate of 90 mm yr^-1^ [22]. The Osa Peninsula is dominated by two main lithological units: the Osa Igneous Complex and the Osa Melange. The Osa Igneous Complex is divided between many subunits comprised mainly of basalt arising from seamounts and oceanic plateaus originating during the middle Eocene to middle Paleocene. The Osa Melange formed after the Igneous Complex, and is comprised of three units originating from acceded mass-wasting deposits, which are overlain with overlap sequences originating from igneous rocks, pelagic sediments as well as shallow water carbonates formed by gravitational and sedimentary processes over the past ~34 MYA. During the closure of Central isthmus between 23–2 MYA, as the volcanic rocks of the present day Osa Peninsula connected with the Chortis Block, subsidence, uplift and oscillations in sea level have caused the peninsula to submerge and re-emerge above sea level several times. The Osa Peninsula has been exposed above sea level for the past two million years.

Our geologic history and lithological maps (Fig 1D) are based on Buchs et al. (2009) [23]. The CAO flight lines covered forests on the Vaquedano and Esquinas subunits within the Osa Igneous Complex, which are dominant on the Osa Peninsula, as well as lesser areas on the Golfito and Volcanic subunits within the much older Golfito Igneous Complex to the east of the Osa Peninsula. The CAO also covered sedimentary sequences formed during the Miocene, Pliocene and Pleistocene, as well as recent Quaternary Alluvium formed through fluvial or erosional processes.

Over time, the combination of high uplift rates [24], which range from 2.5–6.5 m kyr^-1^, and strong erosional forces [25], has yielded a landscape with high topoedaphic diversity (*sensu* [24,26]). The impact of these processes along with climate conditions has driven the geomorphic development of a highly dissected landscape, which is partly mediated by geologic substrata (e.g. [27]). Water basins in the Osa Igenous Complex are much wider, whereas water basins in the Osa Melange Complex are more steeply dissected (see Fig 1). Topographic features, which included slope, terrain ruggedness, convexity, and elevation variance were determined from a CAO LiDAR-based ground digital elevation model (DEM), described later.

Mean annual rainfall varies from 3000–7000 mm across the study region. Here we use data from seven weather stations maintained over five years across the Osa Peninsula to generate a map of mean annual climate conditions across the Osa by interpolation using an inverse distance weighting (IDW) method within the geostatistical toolbox in ArcGIS v.10 (ESRI, Redlands, CA). Mean annual temperature ranges from 24.5–26.5°C, although it did not significantly differ among stations, so temperature was not interpolated alongside precipitation.

### Field plot biomass data

Several groups of researchers have established field inventory plots across the Osa Peninsula, which we use to evaluate our airborne LiDAR-based estimates of forest ACD. The CAO flight lines were collected to maximize coverage of environmental gradients and preexisting plots, which are actively used in ongoing carbon cycling and biodiversity studies. The plots harbor a wide range of biomass levels, which is distributed across highly variable environmental conditions and forest types including successional forests. The lead investigator and details of each contributor’s plot network are briefly described below.

Morales-Salazar et al. [28,29] established 18 0.5-ha plots across the Osa Peninsula as a chronosequence of forests ecosystems of 5, 15, 30 years old secondary as well as several old-growth forest stands. For each plot, the available data included diameter at breast height (dbh; 1.3 m above ground level), tree height and identification to species for all trees ≥ 10 cm dbh, or above the buttress if present. Field data used here were collected in 2011. Hughes et al. (*unpublished*) established three 1.0-ha plots at Piro Biological Station, and the data were collected in 2009 with the same criteria as above. Wanek and Hofhansel (*unpublished*) established 20 1.0-ha plots at five locations across the Osa Peninsula. At each location, four 1-ha plots are located in successional forest, or topographically positioned on a ridge, slope or ravine. Their field data were collected in 2012. Additionally, Weissenhofer et al. [30] established four 1.0-ha plots on a ridge, inland slope, ravine and coastal slope at the Tropical Field Station La Gamba. Altogether, 18 of the above-described plots were covered by the CAO flight lines.

### LiDAR data collection and analysis

The use of LiDAR technology to remotely predict forest biomass is becoming more common (e.g. [15]). LiDAR-based approaches use laser pulses to derive metrics of forest structure in three dimensions (e.g. [14]), which can be validated against field-based estimates of aboveground carbon storage. The LiDAR data were collected using the CAO Airborne Taxonomic Mapping System (AToMS) [16], which includes a dual laser, waveform LiDAR onboard a Dornier 228 aircraft. The LiDAR was operated at 2000 m above ground level (a.g.l.) with a 34° field of view, a pulse repetition frequency of 50 kHz, and a ground speed of approximately 110 kts. The LiDAR has a laser beam divergence set to 0.56 mrad (1/e) for each laser, providing 1.12 m laser spot spacing, and at least two laser shots per m^2^.

Top-of-canopy height (TCH) was estimated by constructing digital surface and ground models [17], and subtracting them to determine vegetation height at 1.12 m resolution. Laser ranges from the LiDAR were combined with embedded high resolution Global Positioning System-Inertial Measurement Unit (GPS-IMU) data to determine the 3-D locations of laser returns, producing a ‘cloud’ of LiDAR data. The LiDAR data cloud consists of a very large number of georeferenced point elevation estimates, where elevation is modeled relative to a reference ellipsoid. The LiDAR data points were processed to identify which laser pulses penetrated the canopy volume and reached the ground surface. We used these points to interpolate a raster digital terrain model (DTM) for the ground surface. This was achieved using a 10 m x 10 m moving kernel to obtain a spatial average ground elevation. Subsequent points were evaluated by fitting a horizontal plane to each of these ground seed points. If the closest unclassified point was < 5.5 m away and < 1.5 m higher in elevation, it was also classified as ground. This process was repeated until all points within the flight coverage were evaluated. The digital surface model (DSM) was based on interpolations of all first-return points. Measurement of the vertical difference between the DTM and DSM yielded the TCH model. In each forest inventory plot, the average of all 1.12 m DCM pixels was used to estimate mean plot TCH as described by Asner and Mascaro [17]. The DTM was used to create a digital elevation model to derive the landscape parameters used in the analysis of topographic effects on ACD variation, such as slope, curvature, elevation, aspect. Previous research has demonstrated the accuracy and reliability of this approach for mapping TCH in tropical forests, including on highly variable terrain such as steep slopes [21, 31].

### Evaluating LiDAR canopy height and above ground carbon density

We compared LiDAR- to field-based estimates of canopy height for 302 individual trees measured with a handheld laser rangefinder (Impulse-200LR, Laser Technology Inc., Englewood, Colorado). To estimate field ACD, we used Model I from Chave et al. [32] as a generalized allometric model to calculate individual tree biomass (AGB) using measured tree height, diameter-at-breast height (dbh) and wood density (ρ), according to:

$${\text{AGB}\;\text{=}\;\text{aD}}^{\text{b1}}{\text{H}}^{\text{b2}}\rho^{\text{b3}\;}$$ (1)

where D is stem diameter (cm), H is canopy height (m) and ρ is wood density (g cm^3^). Within each plot, all individual tree biomass estimates were summed for a plot total. When tree height was not available, we used a diameter-to-height model to estimate tree height [33], and when wood density was not available we used the wood density database by Zanne et al. [34] to estimate AGB by calculating mean wood density (0.56 g per cm3) based on taxonomic values (species, genus or family of the respective tree species composition at the corresponding site (Hofhansl *et al*. *unpublished*). We note that Model II from Chave et al. [32] is well known to over-estimate aboveground biomass or carbon density in most tropical forests [16], and should not be used in humid lowland tropical forests such as the Osa Peninsula, Costa Rica.

To develop a regionally-tuned equation for converting LiDAR TCH to ACD, Asner and Mascaro (2014) prescribed the following based on the concept of plot-aggregate allometry:

$${\text{Estimated}\;\text{ACD}}_{\text{field}}={\text{aTCH}}^{\text{b1}}{\text{BA}}^{\text{b2}}\rho_{\text{BA}}{}^{\text{b3}}$$ (2)

where TCH is LiDAR derived top-of-canopy height, BA is plot-averaged basal area (m^2^ ha) and ρ_BA_ is basal area-weighted wood density. We determined parameters in eq (2) based on a regional calibration technique from Asner and Mascaro [17] using plot-based data described above. The final model parameters for LiDAR-based ACD estimation for Osa forests were *a* = 3.8358, *b*_1_ = 0.2807, *b*_2_ = 0.9721, and *b*_3_ = 1.3763, which are very similar to the parameter values of the universal model developed using hundreds of field sites distributed pantropically [16].

**Fig 2:**
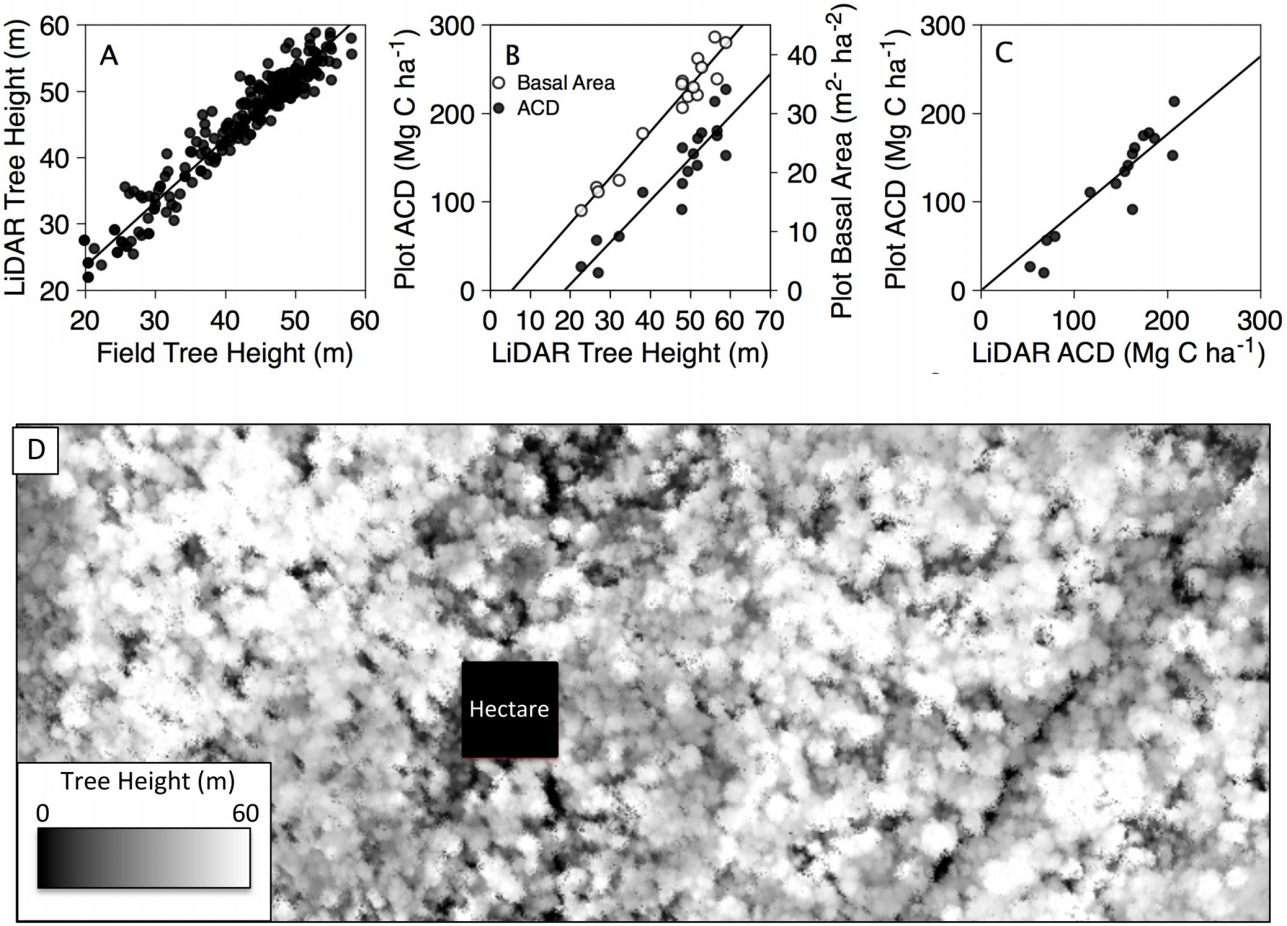
Regional calibration of CAO LiDAR system. (A) Field versus LiDAR measures of individual tree height (y = 0.92x + 6.35, RMSE = 2.49). (B) Plot-level ACD (y = 4.75x - 87.99, RMSE = 22.91) and basal area (y = 0.78x - 4.26, RMSE = 2.25) as a function of LiDAR tree height. The ratio of basal area to LiDAR tree height is the stocking coefficient. (C) Comparison of field versus LiDAR-based estimation of ACD (y = 1.00x - 17.98, RMSE = 16.72). (D) Vegetation canopy height ranging from 0–60 meters after removing variation in topography at 1.12 m spatial resolution. A representative 1-hectare plot, which is the typical size of a forest inventory plot for biomass determination, is shown for spatial comparison.

Next, we mapped ACD by applying eq (2) to maps of LiDAR-derived TCH. Neither BA nor ρ can be directly estimated using LiDAR, so regional relationships with TCH were used to replace these parameters following Asner and Mascaro [17]. Tropical forests display ecoregional differences in height-to-diameter relationships and wood density, and these differences affect comparisons of plot-aggregate allometry to LiDAR metrics. Following Asner and Mascaro [17], we estimated BA in eq (2) using the stocking coefficient, or the linear relationship between TCH and BA using field data from the 18 field plots, where BA = TCH * 0.6767 (Fig 2B). At the plot scale, there was no statistically significant relationship between basal area weighted wood density and TCH (BA = -.0008 *TCH + 0.56, p > 0.05), which we used to factor in a slight wood density adjustment in the final model. The lack of a relationship between wood density and TCH within a floristically-similar region has been explained by Asner et al. [16] and Asner and Mascaro [17]. Altogether, these regional calibrations for stocking coefficient and wood density yielded a final equation for LiDAR-based ACD modeling:

$$\text{ACD}\;=3.8358\;{\left(\text{TCH}\right)}^{0.2807}{\left(TCH\;*\;0.\mathrm{6767}\right)}^{0.9721}{\left(-0.0008\;*\;\text{TCH}\;+\;0.56\right)}^{1.3763}$$ (3)

### Spatial analysis of forest carbon density

Descriptive statistics were derived from the LiDAR-derived DEM for the landscape, including a host of topographic features such as slope, standard deviation of elevation, profile curvature, and the terrain ruggedness index (TRI). We combined these data on topographic heterogeneity with information on climate (e.g. precipitation), geologic substrate, and soil type to examine controls on ACD variation using two multivariate approaches that are useful for analyzing complex spatial data: OLS-SAR (ordinary least squares-simultaneous autoregressive modeling) [35, 36, 37] and CART (Classification and Regression Tree) [38]. These complimentary approaches are useful for examining the underlying sources of variation in a complex spatial dataset with a multitude of possible explanatory variables. The OLS-SAR approach was used to examine the spatial dependence of ACD, which allows the partitioning of variance between the effects of spatial structure independent of environmental gradients and the environmental effects that are independent of spatial structure. However, the partitioning between spatial structure and environmental filtering is limited by the lack of knowledge of potential important environmental variables that are not accounted for here [39,40].

We applied OLS regression with SAR modeling (Lichstein et al. 2002) to a 10% subsample of the data because of the memory-intensiveness of autoregressive analysis [36,37]. To compare results among predictors, all non-binary predictor values were scaled (mean = 0, standard deviation = 0.5). To understand the separate and combined effects of different environmental gradients, geographic trends in biomass levels, and the influence of spatial autocorrelation, we took a multi-stepped approach. First, all possible explanatory variables were combined, and then multicollinearity was minimized if two independent variables were found to have a correlation (Pearson’s R) greater than 0.5. The independent variable that was less correlated to ACD was removed. Independent variables were further eliminated using a reverse stepwise algorithm based on the Akaike information criterion. We used the Moran’s I to test for spatial autocorrelation in the OLS model residuals (P < 0.05), so we computed correlograms to estimate the range for each model (distance at which the variance within the neighborhood equals the overall variance). We selected optimal neighborhood sizes and weightings to minimize spatial autocorrelation of the error term.

In CART analysis, predictor variables were used to recursively partition ACD into categories based on thresholds of each predictor variable, including the total of sum of squares explained by each predictor variable. Optimal tree size was computed using cost-complexity pruning based on 10-fold cross-validation [38] using R 2.11.1 (R development Core team). Both statistical approaches were conducted on 30 and 100 m pixel resolutions, or at 0.09 and 1.0 ha, respectively, as past studies have shown that landscape-scale controls on biomass variation often emerge at these spatial scales [31, 2]. We limited our analysis to areas with primary forest by eliminating zones of land use within the flight coverage.

## Results and Discussion

### Validation of CAO LiDAR measurements of TCH and ACD

The CAO LiDAR system was highly sensitive to variations in forest structure. Field and LiDAR-based measurements of top of canopy height for individual trees were highly correlated across a wide range of tree heights ranging from 20 to 59 m tall (Fig 2A). The CAO LIDAR system was able to measure TCH at 5% error and a reduced mean square error of 2.49 m. The high accuracy of the CAO LiDAR system permitted the measurement and mapping of trees heights ranging from 0 to 67 m tall in forests ranging from less than 15 years of age to primary (old growth) rainforest.

The LiDAR-based measurements of TCH were highly correlated with plot measurements of ACD estimated in inventory plots (Fig 2B, closed circles). The LiDAR metric of TCH predicted plot ACD with 85% accuracy and a reduced mean square error of 22.9 Mg C ha^-1^. This relationship improved when taking into account ecoregional calibrations for both biomass stocking (i.e. the correlation between BA and TCH; Fig 2B, open circles) and wood density using eq (3) above. That is, when biomass stocking and wood density were taken in account, the LiDAR-based approach improved the predictive model to 90% explanatory power (Fig 2C), with a reduced mean squared error of 16.7 Mg C ha^-1^ (Fig 2C). Fig 2C demonstrates that LiDAR and field-based ACD measurements are highly correlated across a range of ACD spanning 25 to 225 Mg C ha^-1^ ACD, with only a slight bias toward over-estimation in LiDAR-estimated ACD at high ACD values.

It’s important to recognize that the residual error in Fig 2C reflects the cumulative errors propagated through both the LiDAR and field based ACD assessments. For example, field measurements of individual tree height are critical for accurate estimation of plot-scale ACD; however, it is difficult to ascertain top-of-canopy height using a handheld laser rangefinder in tall, dense rainforests. Uncertainty is also greatly compounded by the difficulties and assumptions made in measurement techniques and choice of allometric equation [41], as inventory data are processed through allometric models developed by cutting down and measuring the height, mass and density of individual trees from other regions [32]. Also, field plots are typically one hectare in size [41,42] and represent a small fraction of the landscape that can be structurally diverse (Fig 2D). Moreover there is some time offset between CAO and field data collection, which may create a mismatch between airborne and field data. Airborne LiDAR also introduces some error related to laser specifications and settings; however, the TCH metric greatly reduces such errors [43], and makes for a spatially consistent measurement.

Despite these uncertainties, the LiDAR accurately predicted the structure of forests spanning 0 to 67 m in height (see Fig 2D) and 25 to 225 Mg C ha^-1^ACD (Fig 2C). The strong correlation between LiDAR- and field-estimated ACD across a range of forest types demonstrates the efficacy of airborne LiDAR for high-resolution mapping of aboveground carbon stocks in landscapes where the top-of-canopy height reaches 67 m. To date, these are the tallest tropical forests that the CAO has recorded, and contain some of the highest levels of aboveground biomass observed throughout the Neotropics [3, 41,42]. Our findings highlight the importance of this region as a terrestrial carbon reservoir, and demonstrate that airborne LiDAR can be used to accurately estimate ACD in tall, high biomass tropical forests.

### ACD variation across the Osa Peninsula, Costa Rica

ACD varied 9-fold in primary forests across the Osa Peninsula (Fig 2C), from 25 to 225 Mg C ha^-1^. Upland regions with basaltic and sedimentary lithologies exhibited the highest ACD, with emergent trees often exceeding 60 m in height. From a pantropical perspective, Osa rainforests are comparatively larger than most Neotropical rainforests, which typically reach 35–45 m in height [12,17,33], and are more similar to those in Africa and Southeast Asia, which typically reach or exceed 60 m in height [33]. Currently, it’s unclear why primary rainforests on the Osa Peninsula are far larger than the average Neotropical counterpart.

One possible explanation for the larger forests on the Osa Peninsula compared to other well-studied Neotropical regions [42,44] are the comparatively fertile soils ([8, 45, 46], Weintraub et al. *in review*) that support high forest productivity (Wanek et al. 2008). Across the Osa region, soil concentrations of phosphorus, calcium, potassium are generally much higher compared to those reported for Amazonia [47] and other tropical regions [8, 45], where forest productivity is often considered to be limited by bedrock-nutrient availability [48, 49, 50]. High soil fertility across the region likely reflects the joint influence of rapid geologic uplift [24] and weathering [8,25], which jointly rejuvenate soil with bedrock minerals [51]. High soil fertility is reflected in elevated foliar nutrient concentrations [8,11], low canopy nutrient use efficiencies [8] and some of the highest measured rates of net primary production [8] compared to other tropical forests [42,44,52,53]. Under these conditions, it’s plausible that soil fertility underlies that high biomass by enhancing forest productivity, though such processes remain untested and require further experimental research.

Within the Osa Peninsula, as in any ecosystem, parsing controls on forest ACD is complicated because it requires an understanding of how environmental controls influence the balance of stem productivity and mortality over time [10], which are influenced by a host of state variables including species composition, climate, parent material, topography and time. These state factors vary widely across tropical landscapes, and the relative strength and interaction of these controls can shift on local to continental scales [54]. Cognizant of this interplay between factors, we examined how ACD shifts along gradients in geological, topoedaphic and climatic controls. Overall, geologic and topoedaphic factors emerged as the predominant landscape controls on ACD (*discussed below*), though only explained up to 34% of the variation across the Osa Peninsula. Mean annual temperature and rainfall did not emerge as control factors, which could reflect the low temporal and spatial resolution of the data available for this study.

### Geologic Controls on ACD variation

**Fig 3:**
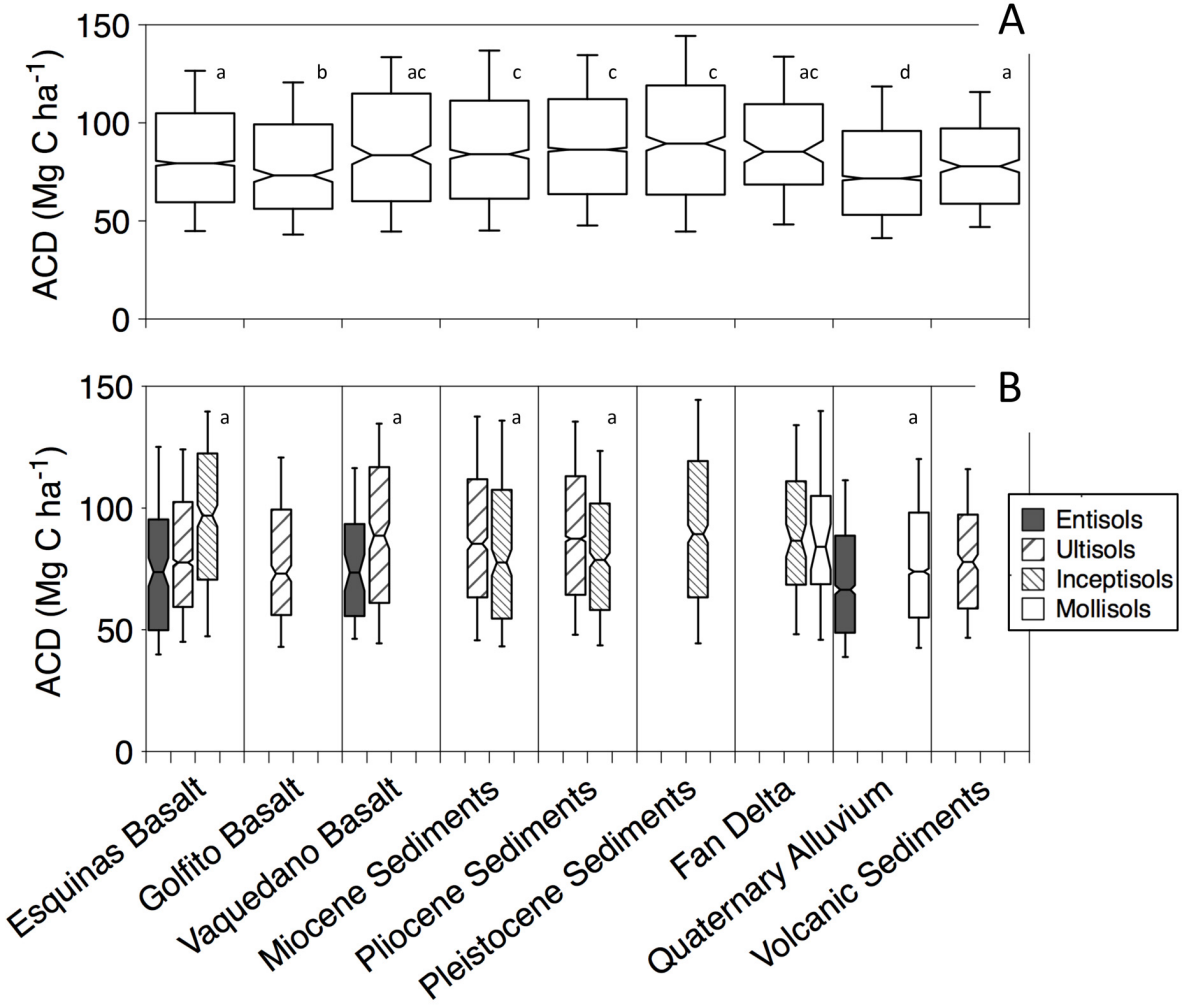
Box-whisker plots showing ACD variation across (A) geologic substrates and (B) soil types. Refer to Fig 1 for visual representation of spatial gradients in geologic orders and soil types.

Geologic substrate played an overarching role in organizing forest biomass patterns at the regional scale. The forests with the highest biomass occur on the high-fertility sedimentary substrates of the Osa Mélange Complex, and secondarily on high-fertility basalt substrata of the Osa Igneous Complex (Fig 3A). Forests on the volcanic sediments, which are much older than the sediments on the Osa Peninsula proper (Fig 1D), harbor less biomass, as did forests on Quaternary Alluvium (Fig 3A).

Quaternary Alluvium is comprised of low-lying fluvial zones or depositional areas of unconsolidated material. Mangroves thrive in the fluvial zones where soils are often waterlogged and rich in organic matter. Such conditions are located primarily in the Rio Sierpe estuary system where fresh and saltwater inundate the land and saturate the soils. Mangrove ACD averaged 69 Mg C ha^-1^ on the Osa Peninsula, which is a typical value for mangroves worldwide [55]. In Corcovado National Park, at the southern end of the peninsula, Quaternary alluvium derives from the erosion of the surrounding sedimentary mountains. These areas are comprised of sandy soils that are nutrient and structurally poor, with adjacent upland areas containing fertile clay-rich soils. There are similar areas in the western Amazon, where low-biomass forests on white sands display far lower biomass because sandy soils offer poor mechanical support for trees, which reduces stem density by increasing tree fall [56,67,58]. These forests stand in stark contrast to the taller neighboring forests growing on upland clay soils, a pattern also observed in the Amazon [56,67,58] and Congo [59] basins. In these scenarios, the relative role of nutrient limitation and soil physical properties is unclear and debated.

Substrate age can have a dramatic effect on forest productivity and carbon storage by regulating soil nutrient abundance [48]. Though Osa’s lithology differs widely in age (> 80 MYA), the influence of parent material age on ACD variation was minimal and only weakly emerged on basalts. That is, ACD is lowest on the oldest Golfito basalts and highest on the younger Vaquedano basalts, but this trend is weak and does not emerge on sedimentary substrates. In the uplands variable geomorphic forces (i.e. uplift, erosion and rainfall) have created a landscape of high topographic diversity and soil complexity. Across multiple geologic substrates on the Osa Peninsula, Bern et al. [60] showed that the majority of mineral nutrients in plant foliage and actively cycling nutrient pools are derived from bedrock sources, suggesting that other factors may dampen the role of substrate age as a regulator on bedrock nutrient availability. Soil type appears to play a subtle role in stratifying ACD within basaltic and sedimentary substrates. For basaltic substrates, ACD generally increased in the order of entisols < ultisols < inceptisols (Fig 3B). For sedimentary substrates, ACD generally increased in the order of entisols < inceptisols < ultisols (Fig 3B). The emergence of soil type as a control likely reflects the underlying role of topographic factors on forest structure, as topographic variation is a leading driver of soil type classifications.

### Topographic controls on ACD variation

**Fig 4:**
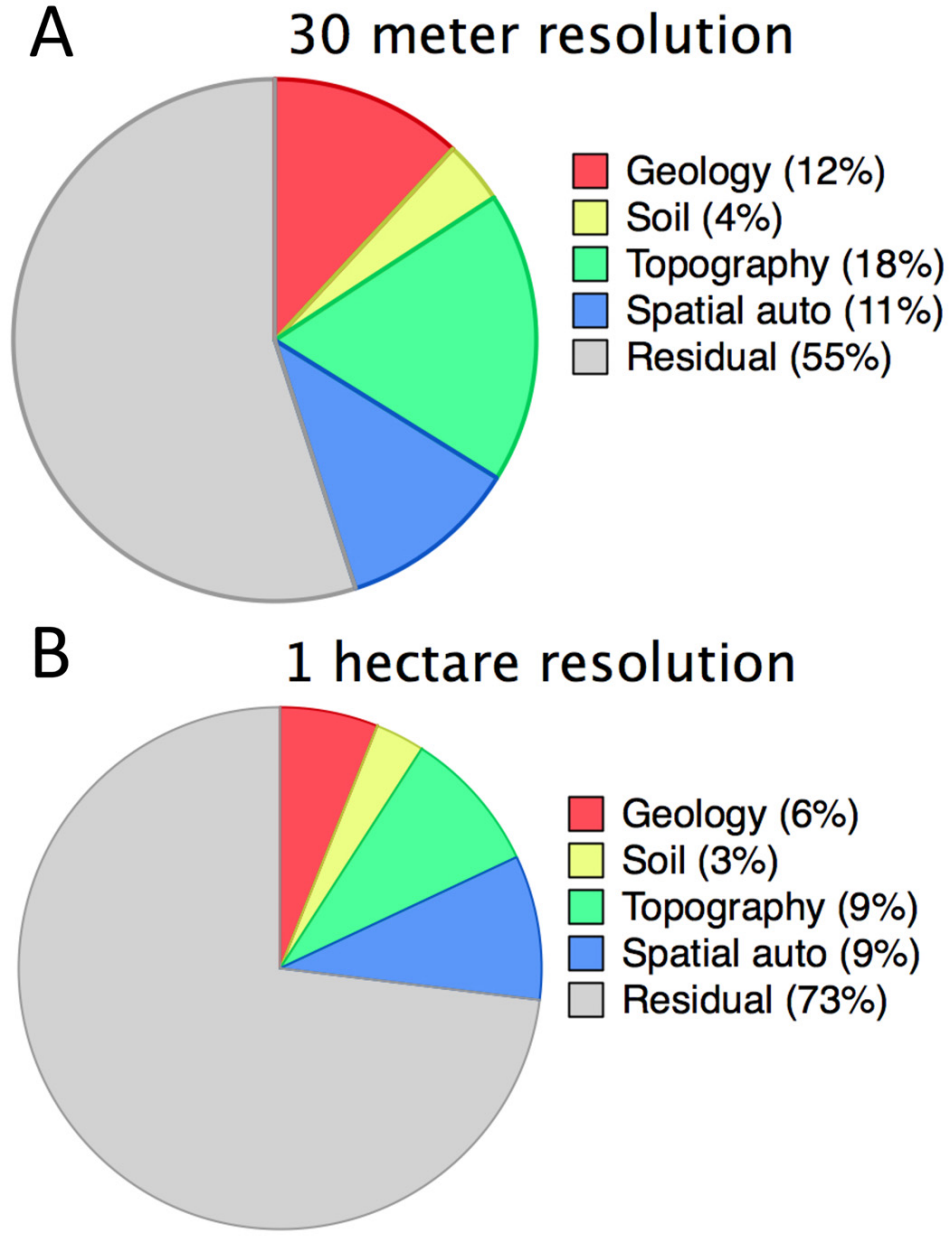
Terrain ruggedness and ACD as a function of elevation for regions underlain with (A) basaltic or (B) sedimentary substrates.

**Fig 5:**
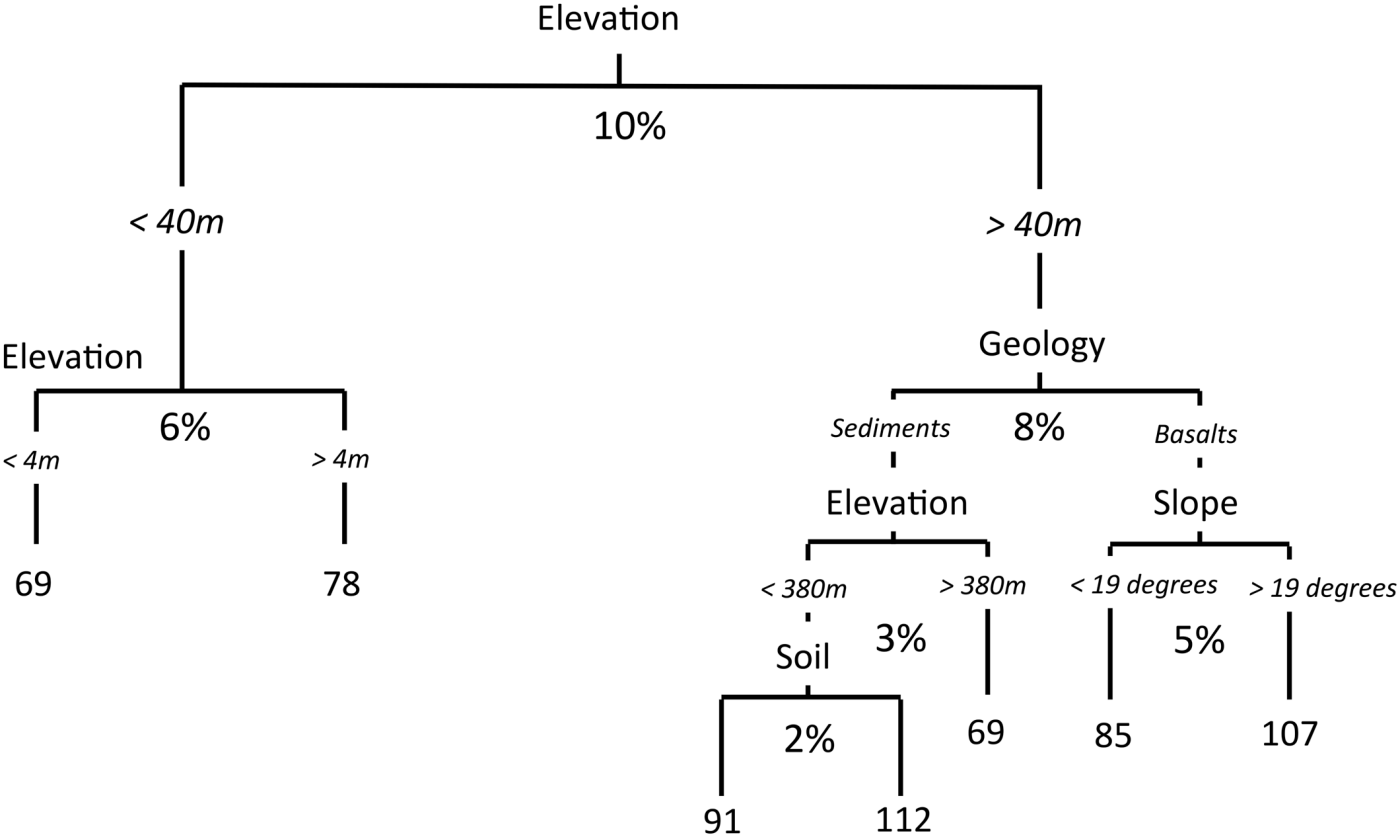
Partitioning of variation among control factors based on the results of SAR-OLS modeling at (A) 30-meter and (B) 1 hectare spatial resolution.

The joint role of topography along with geology was observed in both OLS-SAR and CART modeling. Topographic factors explained 18 and 23% of biomass variation using OLS-SAR (Fig 4) and CART (Fig 5), respectively. Slope was the most important landscape control, explaining 10% of the ACD variation, with terrain ruggedness and curvature each explaining 4% of ACD variation. Elevation emerged as the most important control in CART analysis, dividing upland from lowland regions at a 40 m-elevation threshold (Fig 5). Below 40 m, forests close to sea level had significantly lower ACD, which encompassed forests residing on Quaternary Alluvium discussed above (Fig 5).

**Fig 6:**
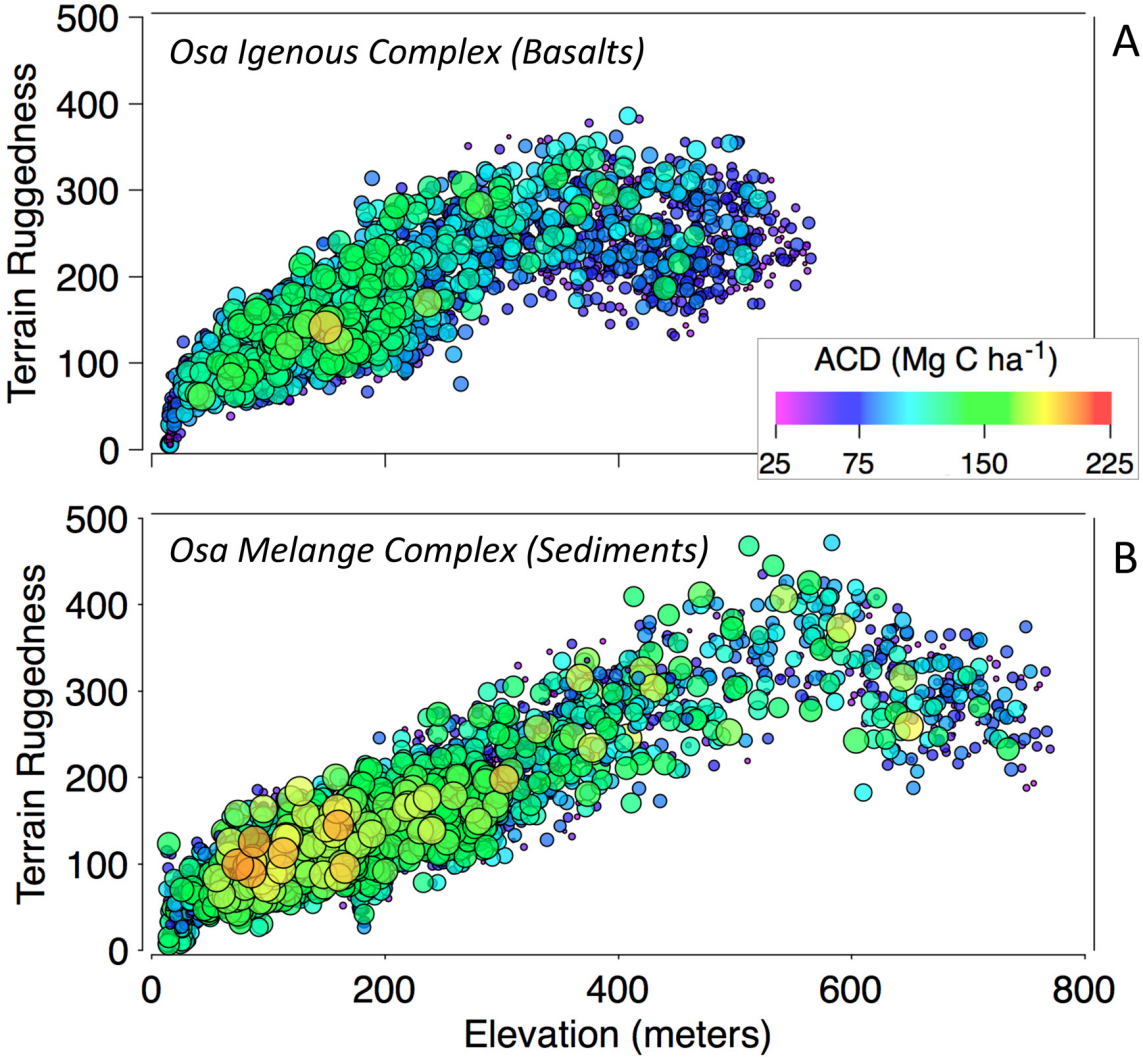
Partitioning of variation among control factors based on the results of CART modeling at 30-meter spatial resolution.

In uplands above 40 m elevation, forests on sedimentary substrates had higher ACD compared to basaltic substrates, which can be observed in Fig 6. ACD declined with increasing elevation in both the Osa Igneous Complex and the Osa Melange; however, ACD decreased substantially on basalts. The decline in ACD with increasing elevation was accompanied by an increase in terrain ruggedness (Fig 6). At intermediate elevations (i.e. 200–400 m), ACD on sedimentary substrate was on average 18% greater compared to forests on basaltic substrata. At the highest elevations, where terrain ruggedness declined (i.e. on the tops of ridges), ACD reached the lowest levels observed among upland forests (Fig 4). The decline in ACD with increasing elevation is widely observed on tropical elevation transects, though the mechanisms are debated (e.g. [61]).

In upland forests, ACD was generally higher on slopes compared to valleys and ridges, which has been observed on soil catenas in the tropics [8, 30, 62, 63]. On basaltic parent material, CART analysis revealed that ACD increased when slope angles exceeded 19° (Fig 5), similar to the slope threshold recently observed on Barro Colorado Island, Panama [21]. Variations in slope affect the formation and chemical properties of soils [26, 48, 51, 62, 64], which arise from differences weathering processes within the landscape. Again, greater soil fertility on slopes could increase ACD by enhancing forest productivity. Indeed, preliminary efforts to quantify topographic differences in soil weathering and nutrient abundance show that ridges and valleys have lower amounts of base cations, in line with the notion that stable environments may be in a more advanced stage of chemical weathering [Wanek & Taylor unpublished data]. Conversely, slopes have greater levels of soil P and higher pH, suggesting a greater role for erosional processes and nutrient redistribution [Weintraub et al. *In* Review, Wanek et al. and Taylor et al. unpublished]. Yet, such speculation warrants more research, as sloping terrain can be unstable and prone to disturbance from landslides and wind throw, which reduce forest biomass in some regions [57, 65, 62].

Multivariate analysis using OLS-SAR revealed a spatial dependence of ACD on environmental factors. That is, landscape factors explained 34% versus 18% of variation in ACD at 30-m and 100-m spatial resolution, respectively (Fig 4A vs. 4B). The higher explanatory power at a 30-m scale suggests that ACD patterns organize on a scale at which landscape terrain variables are changing. The landscape of the Osa Peninsula is steeply dissected, and topography can shift dramatically at small spatial scales. In other regions, such topographic filtering probably depends on regional differences in the spatial scale of geomorphic landscape development. For example, on Barro Colorado Island, Panama, terrain exerts more control on ACD at one hectare than at 30 m scales [21]. In addition, Detto et al. [31] discovered strong fractal similarities in rainforest mean canopy height (a surrogate for ACD; [16,17]) on Barro Colorado Island, with peak correlations between forest structure and topography emerging at scales where edaphic properties change within hydrologic networks.

Forest ACD also displayed spatial dependence separate from the environmental factors considered in our analysis. OLS-SAR modeling revealed that 11% of ACD variation could be explained by spatial autocorrelation, which declined slightly to 9% at 100-m resolution (Fig 4). The neighborhood distances at each scale were similar, 350 and 480 meters at 30 and 100 m, respectively, which indicates that forest structure is more similar within a radius of ~415 m than expected by chance alone. Similar levels and patterns of spatial autocorrelation have been observed elsewhere in the tropics, and could reflect a host of abiotic and biotic processes that regulate forest structure and composition [66,67, 68]. For example, abiotic conditions may lead to phylogenetic or phenotypic clustering, where ecologically similar species assemble with similar functional traits [7, 69]. In the Andes-to-Amazon region, floristic composition, canopy chemistry, forest structure and function are all strongly inter-correlated across gradients of elevation and soil fertility [70]. Biological processes may also contribute to spatial autocorrelation in forest ACD, such as dispersal, stochastic events of colonization and extinction [71,72]. Disentangling the role of evolution from environment is challenging because species attributes reflect the environmental conditions under which they evolved. The relative role and interaction of abiotic (i.e. topoedaphic, lithology, climate) and biotic (i.e. competition, dispersal) processes is still poorly resolved and remains difficult to separate in tropical landscapes where species distribution and diversity is difficult to quantify at scales amenable to empirical evaluation [66, 72].

## Conclusion

Geologic and topographic factors explained up to 34% of the variation in ACD across the Osa Peninsula, Costa Rica, which suggests that the processes of landscape development underlie patterns in forest ACD. Yet, more research is needed to unravel the mechanistic links between soil fertility, topographic diversity and forest C storage. Perhaps a greater mystery to solve is the 66% of ACD variation that remains unexplained by landscape factors. One new way forward in exploring the relative role of environment and phylogeny in regulating forest composition and carbon stocks may rest in the integration of airborne LiDAR with imaging spectroscopy. Trait-based remote sensing using imaging spectroscopy is a promising avenue to using canopy foliar chemical portfolios to understand patterns of species composition and biological diversity [70]. Future research on the Osa Peninsula will focus on integrating data from the new CAO AToMS spectral imaging system [16,17,69] to analyze how spatial variation in forest composition relates to variable levels of forest biomass.

## References

[1] Houghton RA. Aboveground Forest Biomass and the Global Carbon Balance. Global Change Biology. 2005;11: 945–958. 10.1111/j.1365-2486.2005.00955.x

[2] Malhi Y. The productivity, metabolism and carbon cycle of tropical forest vegetation. Journal of Ecology. 2012;100: 65–75. 10.1111/j.1365-2745.2011.01916.x

[3] Gibbs, HK, Brown, S. Geographical Distribution of Woody Biomass Carbon in Tropical Africa: An Updated Database for 2000 [Internet]. 2007 Sep. Available: http://cdiac.ornl.gov/epubs/ndp/ndp055/ndp055b.html

[4] Asner GP, Knapp DE, Broadbent EN, Oliveira PJC, Keller M, Silva JN. Selective Logging in the Brazilian Amazon. Science. 2005;310: 480–482. 10.1126/science.1118051 16239474

[5] Hansen MC, Potapov PV, Moore R, Hancher M, Turubanova SA, Tyukavina A, et al High-Resolution Global Maps of 21st-Century Forest Cover Change. Science. 2013;342: 850–853. 10.1126/science.1244693 24233722

[6] Phillips OL, Aragão LEOC, Lewis SL, Fisher JB, Lloyd J, López-González G, et al Drought Sensitivity of the Amazon Rainforest. Science. 2009;323: 1344–1347. 10.1126/science.1164033 19265020

[7] Quesada CA, Phillips OL, Schwarz M, Czimczik CI, Baker TR, Patiño S, et al Basin-wide variations in Amazon forest structure and function are mediated by both soils and climate. Biogeosciences. 2012;9: 2203–2246. 10.5194/bg-9-2203-2012

[8] Wanek W., Drage S., Hinko N., Hofhansl F., Pölz E.M., Ratzer A. & Richter A. (2008) Primary production and nutrient cycling in lowland rainforests of the Golfo Dulce region In: *Natural and Cultural History of the Golfo Dulce Region*, Costa Rica Biologiezentrum Linz, Stapfia 80, p. 155–178.

[9] Slik JWF, Aiba S-I, Brearley FQ, Cannon CH, Forshed O, Kitayama K, et al Environmental correlates of tree biomass, basal area, wood specific gravity and stem density gradients in Borneo’s tropical forests. Global Ecology and Biogeography. 2010;19: 50–60. 10.1111/j.1466-8238.2009.00489.x

[10] Malhi Y, Wood D, Baker TR, Wright J, Phillips OL, Cochrane T, et al The regional variation of aboveground live biomass in old-growth Amazonian forests. Global Change Biology. 2006;12: 1107–1138. 10.1111/j.1365-2486.2006.01120.x

[11] Townsend AR, Cleveland CC, Asner GP, Bustamante MMC. Controls over foliar n:p ratios in tropical rain forests. Ecology. 2007;88: 107–118. 10.1890/0012-9658(2007)88[107:COFNRI]2.0.CO;2 17489459

[12] Asner GP, Powell GVN, Mascaro J, Knapp DE, Clark JK, Jacobson J, et al High-resolution forest carbon stocks and emissions in the Amazon. PNAS. 2010;107: 16738–16742. 10.1073/pnas.1004875107 20823233 PMC2944749

[13] Saatchi SS, Harris NL, Brown S, Lefsky M, Mitchard ETA, Salas W, et al Benchmark map of forest carbon stocks in tropical regions across three continents. PNAS. 2011; 201019576 10.1073/pnas.1019576108 PMC3116381 21628575

[14] Omasa K, Qiu GY, Watanuki K, Yoshimi K, Akiyama Y. Accurate estimation of forest carbon stocks by 3-D remote sensing of individual trees. Environ Sci Technol. 2003;37: 1198–1201. 12680675 10.1021/es0259887

[15] Gobakken T, Næsset E, Nelson R, Bollandsås OM, Gregoire TG, Ståhl G, et al Estimating biomass in Hedmark County, Norway using national forest inventory field plots and airborne laser scanning. Remote Sensing of Environment. 2012;123: 443–456. 10.1016/j.rse.2012.01.025

[16] Asner GP, Mascaro J, Muller-Landau HC, Vieilledent G, Vaudry R, Rasamoelina M, et al A universal airborne LiDAR approach for tropical forest carbon mapping. Oecologia. 2012;168: 1147–1160. 10.1007/s00442-011-2165-z 22033763

[17] Asner GP, Mascaro J. Mapping tropical forest carbon: Calibrating plot estimates to a simple LiDAR metric. Remote Sensing of Environment. 2014;140: 614–624. 10.1016/j.rse.2013.09.023

[18] Zolkos SG, Goetz SJ, Dubayah R. A meta-analysis of terrestrial aboveground biomass estimation using lidar remote sensing. Remote Sensing of Environment. 2013;128: 289–298. 10.1016/j.rse.2012.10.017

[19] Mitchard ETA, Feldpausch TR, Brienen RJW, Lopez-Gonzalez G, Monteagudo A, Baker TR, et al Markedly divergent estimates of Amazon forest carbon density from ground plots and satellites. Global Ecology and Biogeography. 2014;23: 935–946. 10.1111/geb.12168 26430387 PMC4579864

[20] Colgan MS, Asner GP, Levick SR, Martin RE, Chadwick OA. Topo-edaphic controls over woody plant biomass in South African savannas. Biogeosciences. 2012;9: 1809–1821. 10.5194/bg-9-1809-2012

[21] Mascaro J, Asner GP, Muller-Landau HC, van Breugel M, Hall J, Dahlin K. Controls over aboveground forest carbon density on Barro Colorado Island, Panama. Biogeosciences. 2011;8: 1615–1629. 10.5194/bg-8-1615-2011

[22] Trenkamp R, Kellogg JN, Freymueller JT, Mora HP. Wide plate margin deformation, southern Central America and northwestern South America, CASA GPS observations. Journal of South American Earth Sciences. 2002;15: 157–171. 10.1016/S0895-9811(02)00018-4

[23] Buchs DM, Baumgartner PO, Baumgartner-Mora C, Bandini AN, Jackett S-J, Diserens M-O, et al Late Cretaceous to Miocene seamount accretion and melange formation in the Osa and Burica Peninsulas (Southern Costa Rica): episodic growth of a convergent margin. Geological Society, London, Special Publications. 2009;328: 411–456. 10.1144/SP328.17

[24] Gardner T, Marshall J, Merritts D, Bee B, Burgette R, Burton E, et al Holocene forearc block rotation in response to seamount subduction, southeastern Península de Nicoya, Costa Rica. Geology. 2001;29: 151 10.1130/0091-7613(2001)029<0151:HFBRIR>2.0.CO;2 12067218

[25] Taylor PG, Wieder WR, Weintraub S, Cohen S, Cleveland CC, Townsend AR. Organic forms dominate hydrologic nitrogen export from a lowland tropical watershed. Ecology. 2014;96: 1229–1241. 10.1890/13-1418.1 26236837

[26] Porder S, Chadwick OA. Climate and soil-age constraints on nutrient uplift and retention by plants. Ecology. 2009;90: 623–636. 19341134 10.1890/07-1739.1

[27] Vanwalleghem T, Stockmann U, Minasny B, McBratney AB. A quantitative model for integrating landscape evolution and soil formation. J Geophys Res. 2013;118: 331–347. 10.1029/2011JF002296

[28] Morales-Salazar MS, Vílchez-Alvarado B, Chazdon RL, Ortega-Gutiérrez M, Ortiz-Malavasi E, Guevara-Bonilla M. Diversidad y estructura horizontal en los bosques tropicales del Corredor Biológico de Osa, Costa Rica. Revista Forestal Mesoamericana Kurú. 2012;9: 19–28.

[29] Morales-Salazar MS, Vílchez-Alvarado B, Chazdon RL, Ortiz-Malavasi E, Guevara-Bonilla M. Estructura, composición y diversidad vegetal en bosques tropicales del Corredor Biológico Osa, Costa Rica. Revista Forestal Mesoamericana Kurú. 2013;10: 1–13.

[30] Weissenhofer, A. (2005) Structure and vegetation dynamics of four selected one hectare forest plots in the lowland rain forests of the Piedras Blancas National Park ("Regenwald der Österreicher"), Costa Rica, with notes on the vegetation diversity of the Golfo Dulce region.—PH.D.: University of Vienna.

[31] Detto M, Muller-Landau HC, Mascaro J, Asner GP. Hydrological Networks and Associated Topographic Variation as Templates for the Spatial Organization of Tropical Forest Vegetation. PLoS ONE. 2013;8: e76296 10.1371/journal.pone.0076296 24204610 PMC3799763

[32] Chave J, Andalo C, Brown S, Cairns MA, Chambers JQ, Eamus D, et al Tree allometry and improved estimation of carbon stocks and balance in tropical forests. Oecologia. 2005;145: 87–99. 10.1007/s00442-005-0100-x 15971085

[33] Feldpausch TR, Lloyd J, Lewis SL, Brienen RJW, Gloor M, Monteagudo Mendoza A, et al Tree height integrated into pantropical forest biomass estimates. Biogeosciences. 2012;9: 3381–3403. 10.5194/bg-9-3381-2012

[34] Zanne AE, Lopez-Gonzalez G, Coomes DA, Ilic J, Jansen S, Lewis SL, Miller RB, Swenson NG, Wiemann MC, Chave J. (2009) Global wood density database. Dryad.

[35] Lichstein JW, Simons TR, Shriner SA, Franzreb KE. SPATIAL AUTOCORRELATION AND AUTOREGRESSIVE MODELS IN ECOLOGY. Ecological Monographs. 2002;72: 445–463. 10.1890/0012-9615(2002)072[0445:SAAAMI]2.0.CO;2

[36] Dahlin KM, Asner GP, Field CB. Environmental filtering and land-use history drive patterns in biomass accumulation in a mediterranean-type landscape. Ecological Applications. 2011;22: 104–118. 10.1890/11-1401.1 22471078

[37] Dahlin KM, Asner GP, Field CB. Environmental and community controls on plant canopy chemistry in a Mediterranean-type ecosystem. PNAS. 2013;110: 6895–6900. 10.1073/pnas.1215513110 23569241 PMC3637728

[38] Elith J, Leathwick JR, Hastie T. A working guide to boosted regression trees. Journal of Animal Ecology. 2008;77: 802–813. 10.1111/j.1365-2656.2008.01390.x 18397250

[39] Marmion M, Luoto M, Heikkinen RK, Thuiller W. The performance of state-of-the-art modelling techniques depends on geographical distribution of species. Ecological Modelling. 2009;220: 3512–3520. 10.1016/j.ecolmodel.2008.10.019

[40] Moritz C, Meynard CN, Devictor V, Guizien K, Labrune C, Guarini J-M, et al Disentangling the role of connectivity, environmental filtering, and spatial structure on metacommunity dynamics. Oikos. 2013;122: 1401–1410. 10.1111/j.1600-0706.2013.00377.x

[41] Malhi Y, Aragão LEOC, Metcalfe DB, Paiva R, Quesada CA, Almeida S, et al Comprehensive assessment of carbon productivity, allocation and storage in three Amazonian forests. Global Change Biology. 2009;15: 1255–1274. 10.1111/j.1365-2486.2008.01780.x

[42] Malhi Y, Baker TR, Phillips OL, Almeida S, Alvarez E, Arroyo L, et al The above-ground coarse wood productivity of 104 Neotropical forest plots. Global Change Biology. 2004;10: 563–591. 10.1111/j.1529-8817.2003.00778.x

[43] Mascaro J, Detto M, Asner GP, Muller-Landau HC. Evaluating uncertainty in mapping forest carbon with airborne LiDAR. Remote Sensing of Environment. 2011;115: 3770–3774. 10.1016/j.rse.2011.07.019

[44] Chave J, Navarrete D, Almeida S, Álvarez E, Aragão LEOC, Bonal D, et al Regional and seasonal patterns of litterfall in tropical South America. Biogeosciences. 2010;7: 43–55. 10.5194/bg-7-43-2010

[45] Cleveland CC, Townsend AR, Taylor P, Alvarez-Clare S, Bustamante MMC, Chuyong G, et al Relationships among net primary productivity, nutrients and climate in tropical rain forest: a pan-tropical analysis. Ecology Letters. 2011;14: 939–947. 10.1111/j.1461-0248.2011.01658.x 21749602

[46] Wieder WR, Cleveland CC, Taylor PG, Nemergut DR, Hinckley E-L, Philippot L, et al Experimental removal and addition of leaf litter inputs reduces nitrate production and loss in a lowland tropical forest. Biogeochemistry. 2013;113: 629–642. 10.1007/s10533-012-9793-1

[47] Quesada CA, Lloyd J, Schwarz M, Patiño S, Baker TR, Czimczik C, et al Variations in chemical and physical properties of Amazon forest soils in relation to their genesis. Biogeosciences. 2010;7: 1515–1541. 10.5194/bg-7-1515-2010

[48] Vitousek PM, Porder S, Houlton BZ, Chadwick OA. Terrestrial phosphorus limitation: mechanisms, implications, and nitrogen-phosphorus interactions. Ecol Appl. 2010;20: 5–15. 20349827 10.1890/08-0127.1

[49] Kaspari M, Garcia MN, Harms KE, Santana M, Wright SJ, Yavitt JB. Multiple nutrients limit litterfall and decomposition in a tropical forest. Ecology Letters. 2008;11: 35–43. 10.1111/j.1461-0248.2007.01124.x 18021246

[50] Wright SJ, Yavitt JB, Wurzburger N, Turner BL, Tanner EVJ, Sayer EJ, et al Potassium, phosphorus, or nitrogen limit root allocation, tree growth, or litter production in a lowland tropical forest. Ecology. 2011;92: 1616–1625. 10.1890/10-1558.1 21905428

[51] Porder S, Vitousek PM, Chadwick OA, Chamberlain CP, Hilley GE. Uplift, Erosion, and Phosphorus Limitation in Terrestrial. Ecosystems. 2007;10: 159–171. 10.1007/s10021-006-9011-x

[52] Martinelli LA, Piccolo MC, Townsend AR, Vitousek PM, Cuevas E, McDowell W, et al Nitrogen stable isotopic composition of leaves and soil: Tropical versus temperate forests. Biogeochemistry. 1999;46: 45–65. 10.1023/A:1006100128782

[53] Nardoto GB, Ometto JPHB, Ehleringer JR, Higuchi N, Bustamante MM da C, Martinelli LA. Understanding the Influences of Spatial Patterns on N Availability Within the Brazilian Amazon Forest. Ecosystems. 2008;11: 1234–1246. 10.1007/s10021-008-9189-1

[54] Townsend AR, Asner GP, Cleveland CC. The biogeochemical heterogeneity of tropical forests. Trends in Ecology & Evolution. 2008;23: 424–431. 10.1016/j.tree.2008.04.009 18582987

[55] Komiyama A, Ong JE, Poungparn S. Allometry, biomass, and productivity of mangrove forests: A review. Aquatic Botany. 2008;89: 128–137. 10.1016/j.aquabot.2007.12.006

[56] Phillips OL, Baker TR, Arroyo L, Higuchi N, Killeen TJ, Laurance WF, et al Pattern and process in Amazon tree turnover, 1976–2001. Philos Trans R Soc Lond B Biol Sci. 2004;359: 381–407. 15212092 10.1098/rstb.2003.1438 PMC1693333

[57] Chao K-J, Phillips OL, Gloor E, Monteagudo A, Torres-Lezama A, Martínez RV. Growth and wood density predict tree mortality in Amazon forests. Journal of Ecology. 2008;96: 281–292. 10.1111/j.1365-2745.2007.01343.x

[58] Aguila-Pasquel J del, Doughty CE, Metcalfe DB, Silva-Espejo JE, Girardin CAJ, Gutierrez JAC, et al The seasonal cycle of productivity, metabolism and carbon dynamics in a wet aseasonal forest in north-west Amazonia (Iquitos, Peru). Plant Ecology & Diversity. 2014;7: 71–83. 10.1080/17550874.2013.798365

[59] Kearsley E, de Haulleville T, Hufkens K, Kidimbu A, Toirambe B, Baert G, et al Conventional tree height–diameter relationships significantly overestimate aboveground carbon stocks in the Central Congo Basin. Nat Commun. 2013;4 10.1038/ncomms3269 23912554

[60] Bern CR, Townsend AR, Farmer GL. Unexpected Dominance of Parent-Material Strontium in a Tropical Forest on Highly Weathered Soils. Ecology. 2005;86: 626–632.

[61] Girardin CAJ, Farfan-Rios W, Garcia K, Feeley KJ, Jørgensen PM, Murakami AA, et al Spatial patterns of above-ground structure, biomass and composition in a network of six Andean elevation transects. Plant Ecology & Diversity. 2014;7: 161–171. 10.1080/17550874.2013.820806

[62] Scatena FN, Lugo AE. Geomorphology, disturbance, and the soil and vegetation of two subtropical wet steepland watersheds of Puerto Rico. Available: http://www.treesearch.fs.fed.us/pubs/30266

[63] Takyu M, Aiba S-I, Kitayama K. Changes in Biomass, Productivity and Decomposition along Topographical Gradients under Different Geological Conditions in Tropical Lower Montane Forests on Mount Kinabalu, Borneo. Oecologia. 2003;134: 397–404. 12647148 10.1007/s00442-002-1115-1

[64] Chadwick OA, Roering JJ, Heimsath AM, Levick SR, Asner GP, Khomo L. Shaping post-orogenic landscapes by climate and chemical weathering. Geology. 2013; G34721.1 10.1130/G34721.1

[65] Walker LR; S. The shifting influence of abiotic drivers during landslide succession in Puerto Rico [Internet]. 29 Oct 2013 [cited 27 May 2014]. Available: http://www.nrs.fs.fed.us/pubs/45092

[66] Meynard CN, Lavergne S, Boulangeat I, Garraud L, Van Es J, Mouquet N, et al Disentangling the drivers of metacommunity structure across spatial scales. J Biogeogr. 2013;40: 1560–1571. 10.1111/jbi.12116 24790288 PMC4000944

[67] Fortunel C, Paine CET, Fine PVA, Kraft NJB, Baraloto C. Environmental factors predict community functional composition in Amazonian forests. J Ecol. 2014;102: 145–155. 10.1111/1365-2745.12160

[68] Lebrija-Trejos E, Pérez-García EA, Meave JA, Bongers F, Poorter L. Functional traits and environmental filtering drive community assembly in a species-rich tropical system. Ecology. 2010;91: 386–398. 10.1890/08-1449.1 20392004

[69] Asner GP, Martin RE. Canopy phylogenetic, chemical and spectral assembly in a lowland Amazonian forest. New Phytologist. 2011;189: 999–1012. 10.1111/j.1469-8137.2010.03549.x 21118261

[70] Asner GP, Martin RE, Tupayachi R, Anderson CB, Sinca F, Carranza-Jiménez L, et al Amazonian functional diversity from forest canopy chemical assembly. PNAS. 2014; 201401181 10.1073/pnas.1401181111 PMC3992634 24591585

[71] Holyoak M, Leibold MA, Mouquet NM, Holt RD, Hoopes MF. (2005) Metacommunities: a framework for large-scale community ecology. Metacommunities: spatial dynamics and ecological communities (ed. by Holyoak M, Leibold M.A and Holt R.D), pp. 1–31. University of Chicago Press, Chicago.

[72] Logue JB, Mouquet N, Peter H, Hillebrand H. Empirical approaches to metacommunities: a review and comparison with theory. Trends in Ecology & Evolution. 2011;26: 482–491. 10.1016/j.tree.2011.04.009 21641673

